# LC-MS based sphingolipidomic study on A549 human lung adenocarcinoma cell line and its taxol-resistant strain

**DOI:** 10.1186/s12885-018-4714-x

**Published:** 2018-08-08

**Authors:** Hao Huang, Tian-Tian Tong, Lee-Fong Yau, Cheng-Yu Chen, Jia-Ning Mi, Jing-Rong Wang, Zhi-Hong Jiang

**Affiliations:** 1 State Key Laboratory of Quality Research in Chinese Medicine, Macau Institute for Applied Research in Medicine and Health, Macau University of Science and Technology, Taipa, Macau, China; 2 College of Pharmacy, Gannan Medical University, Ganzhou, 341000 China; 3 International Institute for Translational Chinese Medicine, Guangzhou University of Chinese Medicine, Guangzhou, 510006 China

**Keywords:** A549 human lung adenocarcinoma cell line - Taxol-resistant - LC-MS - Sphingolipids - Ceramide

## Abstract

**Background:**

Resistance to chemotherapy drugs (e.g. taxol) has been a major obstacle in successful cancer treatment. In A549 human lung adenocarcinoma, acquired resistance to the first-line chemotherapy taxol has been a critical problem in clinics. Sphingolipid (SPL) controls various aspects of cell growth, survival, adhesion, and motility in cancer, and has been gradually regarded as a key factor in drug resistance. To better understand the taxol-resistant mechanism, a comprehensive sphingolipidomic approach was carried out to investigate the sphingolipid metabolism in taxol-resistant strain of A549 cell (A549T).

**Methods:**

A549 and A549T cells were extracted according to the procedure with optimal condition for SPLs. Sphingolipidomic analysis was carried out by using an UHPLC coupled with quadrupole time-of-flight (Q-TOF) MS system for qualitative profiling and an UHPLC coupled with triple quadrupole (QQQ) MS system for quantitative analysis. The differentially expressed sphingolipids between taxol-sensitive and -resistant cells were explored by using multivariate analysis.

**Results:**

Based on accurate mass and characteristic fragment ions, 114 SPLs, including 4 new species, were clearly identified. Under the multiple reaction monitoring (MRM) mode of QQQ MS, 75 SPLs were further quantified in both A549 and A549T. Multivariate analysis explored that the levels of 57 sphingolipids significantly altered in A549T comparing to those of A549 (*p* < 0.001 and VIP > 1), including 35 sphingomyelins (SMs), 14 ceramides (Cers), 3 hexosylceramides (HexCers), 4 lactosylceramides (LacCers) and 1 sphingosine. A significant decrease of SM and Cer levels and overall increase of HexCer and LacCer represent the major SPL metabolic characteristic in A549T.

**Conclusions:**

This study investigated sphingolipid profiles in human lung adenocarcinoma cell lines, which is the most comprehensive sphingolipidomic analysis of A549 and A549T. To some extent, the mechanism of taxol-resistance could be attributed to the aberrant sphingolipid metabolism, “inhibition of the de novo synthesis pathway” and “activation of glycosphingolipid pathway” may play the dominant role for taxol-resistance in A549T. This study provides insights into the strategy for clinical diagnosis and treatment of taxol resistant lung cancer.

## Background

Lung cancer has been the leading cause of cancer mortality, and adenocarcinoma is its most prevalent form [1]. Paclitaxel (taxol) is commonly used as part of combination chemotherapy for the treatment of non-small cell lung cancer including adenocarcinoma A549. However, resistance to natural product chemotherapy drugs still constitutes a huge problem of successful cancer treatment, and the efficiency of chemotherapy is weakened because of paclitaxel resistance [2]. Potential mechanisms have been reported including multidrug resistance, β-tubulin alterations, detoxifying of paclitaxel, and apoptosis related genetic changes [3]. Although the extensive efforts have been made for understanding the underlying mechanisms, they are still elusive.

It has been recognized that the dysregulated metabolic profile of cancer is linked to the chemoresistance [4]. Cancer cells reprogram their metabolism to satisfy the demands of malignant phenotype, which decrease drug-induced apoptosis, conferring therapeutic resistance [5]. Since cellular SPLs appear to play a significant role in relation to cancer, their dysregulated synthesis and metabolism in drug-resistant cancer cells have been systematically studied [6]. Most previous studies focus on the biological effect of a kind of specific SPL like Cer [7] and S1P [8] on A549 cancer cell line. The sphingolipid profiles for A549 have been preliminary explored by using MALDI-TOF-MS, only two Cers have been defined as markers out of all the 9 SPLs detected in A549 [9]. The whole sphingolipidome in either A549 or A549T remains largely unrevealed. Recently, a versatile sphingolipidomic approach for both qualitative and quantitative analysis of up to 10 subclasses of SPLs has been established in our group [10]. In this study, the integrated LC-MS approach was employed to investigate the taxol resistance mechanism of A549T from the viewpoint of sphingolipidomic.

## Methods

### Chemicals and materials

The LIPID MAPS internal standard cocktail (internal standards mixture II, 25 μM each of 9 compounds in ethanol, catalog LM-6005) was purchased from Avanti Polar Lipids (Alabaster, AL, USA). It was composed of uncommon SPLs which include: 17-carbon chain length sphingoid base analogs C17-sphingosine [So (d17:1)], C17-sphinganine [Sa (d17:0)], C17-sphingosine-1-phosphate [S1P (d17:1)], C17-sphinganine-1-phosphate [Sa1P (d17:0)], the C12-fatty acid analogs of the more complex SPLs C12-Ceramide [Cer (d18:1/12:0)], C12-ceramide-1-phosphate [C1P (d18:1/12:0)], C12-sphingomyelin [SM (d18:1/12:0)], C12-glucosylceramide [GlcCer (d18:1/12:0)], and C12-lactosylceramide [LacCer (d18:1/12:0)].

Acetic acid (CH_3_COOH, MS grade), formic acid (HCOOH, MS grade), ammonium acetate (NH4OAc, ACS grade) and potassium hydroxide (KOH, ACS grade) were purchased from Sigma-Aldrich (St. Louis, MO, USA). The HPLC grade chloroform (CHCl_3_), isopropanol (IPA), as well as methanol (MeOH) were purchased from Merck (Darmstadt, Germany). Dulbecco’s Modified Eagle’s Medium (DMEM), Roswell Park Memorial Institute (RPMI) 1640 medium, Fetal Bovine Serum (FBS), Penicillin-Streptomycin (PS) were obtained from Gibco, New Zealand. Sodium dodecyl sulfate (SDS) and 3-(4,5-dimethylthiazol-2-yl)-2,5-diphenyltetrazolium bromide (MTT) were acquired from Acros, USA. Ultrapure water (18.2 MΩ) was supplied with a Milli-Q system (Millipore, MA, USA).

### Cell culture and SPLs extraction

A549 human lung adenocarcinoma cell line (Cat.No. KG007) and its taxol-resistant strain (A549T, Cat.No. KG124) were obtained from KeyGen Biotech Co., Ltd. (Nanjing, China). A549 was cultured in DMEM supplemented with 10% FBS and 1% PS in a humidified 5% CO_2_ atmosphere at 37 °C. A549T was cultured in RPMI 1640 medium supplemented with solution consisted of 10% FBS, 1% PS and 200 ng/mL taxol in a humidified 5% CO_2_ atmosphere at 37 °C. For lipid analysis, A549 and A549T cells were respectively seeded into 6-well plates at the density of 1.5 × 10^5^ cells/well and incubated for 48 h. Lipids were extracted from the cells, when they were grown to 80% confluence. After rinsed twice by ice-cold PBS, the cells were scraped into a borosilicate glass tube, in which 0.5 mL of MeOH, 0.25 mL of CHCl_3_ and 10 μL of 2.5 μM internal standards cocktail were added. The extract procedure was carried out by incubation at 48 °C for 12 h after sonicated at ambient temperature for 30 s. After 75 μL of KOH in MeOH (1 M) was added, the mixture was placed into a shaking incubator at 37 °C for 2 h. Acetic acid was used to neutralize the mixture before the typical four-step extraction was carried out for the preparation of SPLs. Further details for extracting SPLs and sample preparation were the same as previously described [11]. MTT assay was employed to evaluate the sensitivity of A549 and A549T cells to taxol. The IC_50_s were 67.72 nM and 124.7 μM, respectively corresponding to A549 and A549T, showing almost 2000-fold difference in taxol sensitivity between these two cell lines.

### LC-MS conditions

Sphingolipid analysis was performed by using our developed LC-MS method with minor optimization, just as described previously [10, 11]. Chromatographic separation was achieved by using an Agilent 1290 UHPLC system, and it was interfaced with an Agilent ultrahigh definition 6550 Q-TOF mass spectrometer and an Agilent 6460 triple-quadrupole mass spectrometer respectively for qualitative- and quantitative-analysis. The acquisition and data analysis were operated by using Agilent MassHunter Workstation Software.

### Data analysis

Based on the Agilent Personal Compound Database and Library (PCDL) software and LIPID MAPS Lipidomics Gateway, a personal database has been established with the latest update of 32,622 SPLs until August 06 2016. The screening and identification of SPLs were carried out by searching against it.

In qualitative research, the sphingolipidomic approach was applied by analyzing QC samples equally pooled by A549 and A549T. In quantitative research, A549 cells (models, *n* = 10) and A549T cells (models, *n* = 10), as well as QC samples (*n* = 5), were analyzed in parallel. Multivariate statistical analysis, including principle component analysis (PCA) and partial least squares to latent structure-discriminant analysis (PLS-DA) methods, were performed to examine significant differences between A549 and A549T, using SIMCA-P+ software version 14.0 (Umetrics, Umea, Sweden). Variable Importance in the Project (VIP) value in PLS-DA model was used for selecting and identifying biomarkers. The altered SPL with a VIP value larger than 1.00 was considered as a biomarker.

## Results

### Comprehensive profiling of sphingolipids in A549 and A549T cells

QC samples were analyzed repeatedly to achieve comprehensive profiling of SPLs in A549 and A549T. In various subclasses of SPLs, the [M + H]^+^ ions exhibits highest intensities in positive ion mode. Totally 114 SPLs have been identified in the QC samples, among which Cer (d18:2/26:2), DHCer (d18:0/24:2), phytosphingosine (PTSo) t19:2, and PTSo t16:1 were new SPLs. Notably, 4 pairs of isobaric species (**A**_**1**_**-A**_**4**_ vs **a**_**1**_**-a**_**4**_) and 21 pairs of isomeric species (**B**_**1**_**-B**_**21**_ vs **b**_**1**_**-b**_**21**_) were clearly distinguished in this study. Respective qualitative test of A549 and A549T revealed that they share all the same species of SPLs. The full identification result was listed in Table 1.

**Table 1:** Identification and quantification of SPLs in A549/A549T cells by using UHPLC-Q-TOF and UHPLC-QQQ MS

Class	Name	[M + H]^+^ *m*/*z*	t_R_ (min)	Molecular Formula	Measured Mass	Calculated Mass	Error (ppm)	MS/MS Fragments (*m*/*z*)	MRM transitions	
SM	d18:1/26:0 **[B**_**1**_**]**	843.7314	18.483	C_49_ H_99_ N_2_ O_6_ P	842.7240	842.7241	−0.12	264.2674, 184.0730	843.7	184.1
	d18:1/26:1	841.7096	17.136	C_49_ H_97_ N_2_ O_6_ P	840.7026	840.7084	−6.90	264.2682, 184.0739	841.7	184.1
	d18:1/25:0 **[B**_**2**_**]**	829.7154	17.702	C_48_ H_97_ N_2_ O_6_ P	828.7080	828.7084	−0.48	264.2683, 184.0732	829.7	184.1
	d18:1/25:1 **[B**_**3**_**]**	827.6993	16.288	C_48_ H_95_ N_2_ O_6_ P	826.6915	826.6928	−1.57	264.2698, 184.0722	827.7	184.1
	d18:1/24:0 **[B**_**4**_**]**	815.6992	17.020	C_47_ H_95_ N_2_ O_6_ P	814.6925	814.6928	−0.37	264.2697, 184.0732	815.7	184.1
	d18:1/24:1 **[B**_**5**_**]**	813.6841	15.507	C_47_ H_93_ N_2_ O_6_ P	812.6769	812.6771	−0.25	264.2685, 184.0739	813.7	184.1
	d18:1/24:2	811.6680	14.958	C_47_ H_91_ N_2_ O_6_ P	810.6607	810.6615	−0.99	264.2688, 184.0732	811.7	184.1
	d18:1/24:3 **[B**_**6**_**]**	809.6526	14.243	C_47_ H_89_ N_2_ O_6_ P	808.6459	808.6458	0.12	264.2627, 184.0725	809.7	184.1
	d18:1/23:0 **[B**_**7**_**]**	801.6839	16.371	C_46_ H_93_ N_2_ O_6_ P	800.6769	800.6771	−0.25	264.2645, 184.0727	801.7	184.1
	d18:1/23:1 **[B**_**8**_**]**	799.6683	15.241	C_46_ H_91_ N_2_ O_6_ P	798.6608	798.6615	−0.88	264.2654, 184.0721	799.7	184.1
	d18:1/23:2	797.6520	14.326	C_46_ H_89_ N_2_ O_6_ P	796.6443	796.6458	−1.88	264.2398, 184.0727	797.7	184.1
	d18:1/22:0 **[B**_**9**_**]**	787.6679	15.723	C_45_ H_91_ N_2_ O_6_ P	786.6608	786.6615	−0.89	264.2665, 184.0730	787.7	184.1
	d18:1/22:1 **[B**_**10**_**]**	785.6524	14.642	C_45_ H_89_ N_2_ O_6_ P	784.6452	784.6458	−0.76	264.2606, 184.0730	785.7	184.1
	d18:1/22:2	783.6362	13.728	C_45_ H_87_ N_2_ O_6_ P	782.6291	782.6302	−1.41	264.2636, 184.0727	783.6	184.1
	d18:1/21:0	773.6528	15.108	C_44_ H_89_ N_2_ O_6_ P	772.6455	772.6458	−0.39	264.2651, 184.0721	773.7	184.1
	d18:1/21:1 **[B**_**11**_**]**	771.6361	14.010	C_44_ H_87_ N_2_ O_6_ P	770.6286	770.6302	−2.08	264.2624, 184.0732	771.6	184.1
	d18:1/20:0	759.6368	14.459	C_43_ H_87_ N_2_ O_6_ P	758.6295	758.6302	−0.92	264.2658, 184.0730	759.6	184.1
	d18:1/20:1 **[B**_**12**_**]**	757.6201	13.428	C_43_ H_85_ N_2_ O_6_ P	756.6128	756.6145	−2.25	264.2653, 184.0735	757.6	184.1
	d18:1/19:0	745.6207	13.811	C_42_ H_85_ N_2_ O_6_ P	744.6134	744.6145	−1.48	264.2677, 184.0725	745.6	184.1
	d18:1/18:0	731.6054	13.195	C_41_ H_83_ N_2_ O_6_ P	730.5982	730.5989	−0.96	264.2665, 184.0727	731.6	184.1
	d18:1/18:1 **[B**_**13**_**]**	729.5869	12.081	C_41_ H_81_ N_2_ O_6_ P	728.5786	728.5832	−8.23	264.2659, 184.0732	729.6	184.1
	d18:1/17:0	717.5896	12.613	C_40_ H_81_ N_2_ O_6_ P	716.5824	716.5832	−1.12	264.2677, 184.0726	717.6	184.1
	d18:1/16:0	703.5737	12.081	C_39_ H_79_ N_2_ O_6_ P	702.5666	702.5676	−1.42	264.2680, 184.0748	703.6	184.1
	d18:1/16:1 **[B**_**14**_**]**	701.5583	11.366	C_39_ H_77_ N_2_ O_6_ P	700.5511	700.5519	−1.14	264.2685, 184.0730	701.6	184.1
	d18:1/15:0	689.5585	11.616	C_38_ H_77_ N_2_ O_6_ P	688.5512	688.5519	−1.02	264.2627, 184.0726	689.6	184.1
	d18:1/15:1 **[B**_**15**_**]**	687.5420	10.752	C_38_ H_75_ N_2_ O_6_ P	686.5352	686.5363	−1.60	264.2629, 184.0720	687.5	184.1
	d18:1/14:0	675.5427	11.117	C_37_ H_75_ N_2_ O_6_ P	674.5355	674.5363	−1.19	264.2686, 184.0734	675.5	184.1
	d18:2/25:0 **[b**_**3**_**]**	827.6988	16.504	C_48_ H_95_ N_2_ O_6_ P	826.6896	826.6928	−3.87	262.2524, 184.0729		
	d18:2/24:0 **[b**_**5**_**]**	813.6763	15.873	C_47_ H_93_ N_2_ O_6_ P	812.6693	812.6771	−9.60	262.2542, 184.0726		
	d18:2/24:2 **[b**_**6**_**]**	809.6503	15.723	C_47_ H_89_ N_2_ O_6_ P	808.6457	808.6458	−0.12	262.2554, 184.0731		
	d18:2/24:3	807.6343	14.659	C_47_ H_87_ N_2_ O_6_ P	806.6272	806.6302	−3.71	184.0725		
	d18:2/23:0 **[b**_**8**_**]**	799.6684	15.474	C_46_ H_91_ N_2_ O_6_ P	798.6606	798.6615	−1.13	184.0732	799.7	184.1
	d18:2/22:0 **[b**_**10**_**]**	785.6523	14.808	C_45_ H_89_ N_2_ O_6_ P	784.6451	784.6458	−0.89	262.2440, 184.0732	785.7	184.1
	d18:2/21:0 **[b**_**11**_**]**	771.6357	14.193	C_44_ H_87_ N_2_ O_6_ P	770.6272	770.6302	−3.89	184.0722		
	d18:2/20:0 **[b**_**12**_**]**	757.6200	13.545	C_43_ H_85_ N_2_ O_6_ P	756.6128	756.6145	−2.25	262.2451, 184.0725	757.6	184.1
	d18:2/18:0 **[b**_**13**_**]**	729.5896	12.347	C_41_ H_81_ N_2_ O_6_ P	728.5822	728.5832	−1.37	262.2554, 184.0728		
	d18:2/16:0 **[b**_**14**_**]**	701.5582	11.382	C_39_ H_77_ N_2_ O_6_ P	700.5510	700.5519	−1.28	262.2504, 184.0728		
	d18:2/15:0 **[b**_**15**_**]**	687.5433	10.951	C_38_ H_75_ N_2_ O_6_ P	686.5355	686.5363	−1.16	262.2503, 184.0716		
	d18:1/12:0 **[IS-1]**	647.5116	10.402	C_35_ H_71_ N_2_ O_6_ P	646.5042	646.5050	−1.24	264.2699, 184.0732	647.5	184.1
DHSM	d18:0/26:0	845.7455	19.182	C_49_ H_101_ N_2_ O_6_ P	844.7382	844.7397	−1.78	266.2711, 184.0730		
	d18:0/26:1 **[b**_**1**_**]**	843.7271	17.702	C_49_ H_99_ N_2_ O_6_ P	842.7202	842.7241	−4.63	266.2787, 184.0727		
	d18:0/25:0	831.7297	18.317	C_48_ H_99_ N_2_ O_6_ P	830.7224	830.7241	−2.05	184.0731		
	d18:0/25:1 **[b**_**2**_**]**	829.7149	17.469	C_48_ H_97_ N_2_ O_6_ P	828.7071	828.7084	−1.57	266.2729, 184.0729		
	d18:0/24:0	817.7151	17.585	C_47_ H_97_ N_2_ O_6_ P	816.7081	816.7084	−0.37	266.2696, 184.0732	817.7	184.1
	d18:0/24:1 **[b**_**4**_**]**	815.6992	16.405	C_47_ H_95_ N_2_ O_6_ P	814.6931	814.6928	0.37	266.2767, 184.0732		
	d18:0/23:0	803.6995	16.937	C_46_ H_95_ N_2_ O_6_ P	802.6921	802.6928	−0.87	184.0725	803.7	184.1
	d18:0/23:1 **[b**_**7**_**]**	801.6842	15.756	C_46_ H_93_ N_2_ O_6_ P	800.6767	800.6771	−0.50	184.0728	801.7	184.1
	d18:0/22:0	789.6838	16.272	C_45_ H_93_ N_2_ O_6_ P	788.6771	788.6771	0.00	184.0129	789.7	184.1
	d18:0/22:1 **[b**_**9**_**]**	787.6684	15.141	C_45_ H_91_ N_2_ O_6_ P	786.6612	786.6615	−0.38	184.0731		
	d18:0/21:0	775.6672	15.640	C_44_ H_91_ N_2_ O_6_ P	774.6598	774.6615	−2.19	184.0728		
	d18:0/20:0	761.6528	14.958	C_43_ H_89_ N_2_ O_6_ P	760.6452	760.6458	−0.79	184.0728	761.7	184.1
	d18:0/19:0	747.6355	14.326	C_42_ H_87_ N_2_ O_6_ P	746.6312	746.6302	1.34	184.0728	747.6	184.1
	d18:0/18:0	733.6210	13.694	C_41_ H_85_ N_2_ O_6_ P	732.6140	732.6145	−0.68	184.0730	733.6	184.1
	d18:0/17:0	719.6056	13.096	C_40_ H_83_ N_2_ O_6_ P	718.5982	718.5989	−0.97	266.2542, 184.0730	719.6	184.1
	d18:0/16:0	705.5896	12.514	C_39_ H_81_ N_2_ O_6_ P	704.5823	704.5832	−1.28	184.0739	705.6	184.1
	d18:0/15:0	691.5737	11.982	C_38_ H_79_ N_2_ O_6_ P	690.5666	690.5676	−1.45	184.0729	691.6	184.1
	d18:0/14:0	677.5586	11.466	C_37_ H_77_ N_2_ O_6_ P	676.5512	676.5519	−1.03	266.2797, 184.0729	677.6	184.1
Cer	d18:1/26:0	678.6745	20.495	C_44_ H_87_ N O_3_	677.6679	677.6686	−1.03	264.2681		
	d18:1/25:0	664.6584	19.481	C_43_ H_85_ N O_3_	663.6526	663.6529	−0.45	264.2654		
	d18:1/24:0 **[B**_**16**_**]**	650.6436	18.566	C_42_ H_83_ N O_3_	649.6365	649.6373	−1.23	264.2652	650.6	264.3
	d18:1/24:1 **[B**_**17**_**]**	648.6280	17.219	C_42_ H_81_ N O_3_	647.6208	647.6216	−1.24	264.2681	648.6	264.3
	d18:1/23:0	636.6272	17.070	C_41_ H_81_ N O_3_	635.6251	635.6216	5.51	264.2681	636.6	264.3
	d18:1/23:1	634.6120	16.588	C_41_ H_79_ N O_3_	633.6045	633.6060	−2.37	264.2685	634.6	264.3
	d18:1/22:0 **[B**_**18**_**]**	622.6124	17.086	C_40_ H_79_ N O_3_	621.6050	621.6060	−1.61	264.2680	622.6	264.3
	d18:1/22:1 **[B**_**19**_**]**	620.5964	15.956	C_40_ H_77_ N O_3_	619.5891	619.5903	−1.94	264.2681	620.6	264.3
	d18:1/20:0	594.5805	15.706	C_38_ H_75_ N O_3_	593.5732	593.5747	−2.53	264.2686	594.6	264.3
	d18:1/18:0	566.5497	14.376	C_36_ H_71_ N O_3_	565.5423	565.5434	−1.95	264.2673	566.5	264.3
	d18:1/18:1	564.5302	13.478	C_36_ H_69_ N O_3_	563.5233	563.5278	0.53	264.2669	563.5	264.3
	d18:1/17:0	574.5155	13.744	C_35_ H_69_ N O_3_	551.5259	551.5277	−3.26	264.2670		
	d18:1/16:0	538.5186	13.112	C_34_ H_67_ N O_3_	537.5114	537.5121	−1.30	264.2683	538.5	264.3
	d18:1/16:1 **[B**_**20**_**]**	536.5028	12.298	C_34_ H_65_ N O_3_	535.4952	535.4964	−2.24	264.2681	536.5	264.3
	d18:1/15:0	524.5026	12.564	C_33_ H_65_ N O_3_	523.4955	523.4964	−1.72	264.2659	524.5	264.3
	d18:1/14:0	510.4868	11.982	C_32_ H_63_ N O_3_	509.4793	509.4808	−2.94	264.2696		
	d18:2/26:2	672.6258	18.566	C_44_ H_81_ N O_3_	671.6185	671.6216	−4.62	262.2530	672.6	262.3
	d18:2/24:1	646.6121	16.288	C_42_ H_79_ N O_3_	645.6047	645.6060	−2.01	262.2528	646.6	262.3
	d18:2/22:0 **[b**_**19**_**]**	620.5960	16.139	C_40_ H_77_ N O_3_	619.5886	619.5903	−2.74	262.2528	620.6	262.3
	d18:2/16:0 **[b**_**20**_**]**	536.5029	12.554	C_34_ H_65_ N O_3_	535.4953	535.4964	−2.05	262.2532		
	d18:1/12:0 **[IS-2]**	482.4556	11.034	C_30_ H_59_ N O_3_	481.4479	481.4495	−3.32	264.2678	482.5	264.3
DHCer	d18:0/24:0	652.6587	19.198	C_42_ H_85_ N O _3_	651.6513	651.6529	−2.46	266.2853	652.7	266.3
	d18:0/24:1 **[b**_**16**_**]**	650.6434	17.735	C_42_ H_83_ N O_3_	649.6361	649.6373	−1.85	266.2837		
	d18:0/24:2 **[b**_**17**_**]**	648.6281	17.502	C_42_ H_81_ N O_3_	647.6209	647.6216	−1.08	266.2826		
	d18:0/22:0	624.6277	17.569	C_40_ H_81_ N O_3_	623.6202	623.6216	−1.12	266.2859		
	d18:0/22:1 **[b**_**18**_**]**	622.6111	16.438	C_40_ H_79_ N O_3_	621.6036	621.6060	−3.86	266.2779		
	d18:0/20:0	596.5956	16.222	C_38_ H_77_ N O_3_	595.5878	595.5903	−4.20	266.2811		
	d18:0/18:0	568.5650	14.858	C_36_ H_73_ N O_3_	567.5578	567.5590	−2.11	266.2844		
	d18:0/16:0	540.5343	13.561	C_34_ H_69_ N O_3_	539.5272	539.5277	−0.93	266.2822		
PTCer	t18:0/14:0	528.4981	11.333	C_32_ H_65_ N O_4_	527.4908	527.4914	−1.14	514.4823, 264.2687		
HexCer	d18:1/26:0	840.7273	18.467	C_50_ H_97_ N O_8_	839.7189	839.7214	− 2.98	264.2685	840.7	264.3
	d18:1/24:0	812.6974	17.020	C_48_ H_93_ N O_8_	811.6893	811.6901	−0.99	264.2685	812.7	264.3
	d18:1/24:1	810.6809	16.654	C_48_ H_91_ N O_8_	809.6732	809.6745	−1.61	264.2677	810.7	264.3
	d18:1/23:0	798.6804	16.388	C_47_ H_91_ N O_8_	797.6725	797.6745	−2.51	264.2676	798.7	264.3
	d18:1/22:0	784.6656	15.740	C_46_ H_89_ N O_8_	783.6579	783.6588	−1.15	264.2689	784.7	264.3
	d18:1/16:0	700.5717	12.115	C_40_ H_77_ N O_8_	699.5621	699.5649	−4.00	264.2689	700.6	264.3
	d18:1/12:0 **[IS-3]**	644.5101	10.435	C_36_ H_69_ N O_8_	643.5007	643.5023	−2.49	264.2684	644.5	264.3
LacCer	d18:1/24:0	974.7508	16.388	C_54_ H_103_ N O_13_	973.7432	973.7429	0.31	264.2672	974.7	264.3
	d18:1/24:1	972.7329	15.257	C_54_ H_101_ N O_13_	971.7253	971.7273	−2.06	264.2650	972.7	264.3
	d18:1/22:0	946.7190	15.124	C_52_ H_99_ N O_13_	945.7112	945.7116	−0.42	264.2679	946.7	264.3
	d18:1/20:0	918.6866	13.894	C_50_ H_95_ N O_13_	917.6780	917.6803	−2.51	264.2688		
	d18:1/18:0	890.6552	12.713	C_48_ H_91_ N O_13_	889.6469	889.6490	−2.36	264.2696	890.7	264.3
	d18:1/16:0	862.6250	11.682	C_46_ H_87_ N O_13_	861.6175	861.6177	−0.23	264.2687	862.6	264.3
	d18:1/12:0 **[IS-4]**	806.5623	10.219	C_42_ H_79_ N O_13_	805.5550	805.5551	−0.13	264.2683	806.7	264.3
Sa	d19:0 **[A**_**1**_**]**	316.3202	6.532	C_19_ H_41_ N O_2_	315.3135	315.3137	−0.63	298.3106, 272.2906	274.3	256.3
	d18:0 **[B**_**21**_**] [A**_**2**_**]**	302.3050	6.993	C_18_ H_39_ N O_2_	301.2972	301.2981	−2.98	284.2921	302.3	284.3
	d16:0	274.2739	4.881	C_16_ H_35_ N O_2_	273.2666	273.2668	0.73	256.2627	274.3	256.3
PTSa	t17:0	304.2874	5.468	C_17_ H_37_ N O_3_	303.2782	303.2773	2.68	286.2751		
	d17:0 **[A**_**3**_**] [IS-5]**	288.2901	6.632	C_17_ H_37_ N O_2_	287.2829	287.2824	1.65	270.2794	288.3	270.3
So	d19:1	314.3051	10.668	C_19_ H_39_ N O_2_	313.2978	313.2981	−0.96	296.3320		
	d18:1	300.2896	6.760	C_18_ H_37_ N O_2_	299.2822	299.2824	−0.67	282.2787, 264.2689	300.3	282.3
	d17:1 **[A**_**4**_**]**	286.2737	6.444	C_17_ H_35_ N O_2_	285.2659	285.2668	−3.15	270.2783		
	d16:1	272.2582	5.264	C_16_ H_33_ N O_2_	271.2505	271.2511	−2.36	254.2833	272.3	254.3
	d15:1	258.2425	6.711	C_15_ H_31_ N O_2_	257.2347	257.2355	−3.11	240.2319		
	d15:2	256.2270	5.064	C_15_ H_29_ N O_2_	255.2192	255.2198	−2.35	238.2214		
PTSo	t19:1	330.3003	8.041	C_19_ H_39_ N O_3_	329.2954	329.2930	7.29	312.3278		
	t19:2	328.2846	6.245	C_19_ H_37_ N O_3_	327.2771	327.2773	−0.61	310.2996		
	t18:1 **[a**_**1**_**]**	316.2850	6.874	C_18_ H_37_ N O_3_	315.2739	315.2773	10.8	298.2739, 280.2632, 262.2522	316.3	298.3
	t17:1 **[a**_**2**_**]**	302.2687	7.176	C_17_ H_35_ N O_3_	301.2618	301.2617	0.33	284.2921, 266.2838	302.3	284.3
	t16:1 **[a**_**3**_**]**	288.2537	5.663	C_16_ H_33_ N O_3_	287.2459	287.2460	−0.35	270.2786		
	d17:1 **[a**_**4**_**] [IS-6]**	286.3106	6.558	C_18_ H_39_ N O	285.3034	285.3032	0.74	268.2643	286.3	268.3
SBA	Enigmol **[b**_**21**_**] [A**_**2**_**]**	302.3052	5.081	C_18_ H_39_ N O_2_	301.2974	301.2981	−2.32	284.2930, 266.2090		
SBA	Xestoaminol C	230.2477	5.131	C_14_ H_31_ N O	229.2404	229.2406	−0.87	212.2355		
C1P	d18:1/12:0 **[IS-7]**	562.4223	10.006	C_30_ H_60_ N O_6_ P	561.4149	561.4158	−1.72	264.2688	562.5	264.3
Sa1P	d17:0 **[IS-8]**	368.2574	6.774	C_17_ H_38_ N O_5_ P	367.2504	367.2488	4.57		368.3	270.3
So1P	d17:1 **[IS-9]**	366.2406	6.558	C_17_ H_36_ N O_5_ P	365.2331	365.2331	0.03	250.2510	366.2	250.3

The sphingolipids are classified according to “lipid classification system” (http://www.lipidmaps.org/)

*SM* sphingomyelin, *DHSM* dihydrosphingomyelin, *Cer* Ceramide, *DHCer* dihydroceramide, *PTCer* phytoceramides, *HexCer* hexosylceramide, *LacCer* lactosylceramide, *Sa* sphinganine, *PTSa* phytosphinganine, *So* sphingosine, *PTSo* phytosphingosine, *SBA* sphingoid base analog, *C1P* ceramide-1-phosphate, *Sa1P* sphinganine-1-phosphate, *So1P* sphingosine-1-phosphate

**[A**_**1**_**-A**_**4**_ **vs a**_**1**_**-a**_**4**_**]** 4 pairs of isomeric sphingolipids; **[B**_**1**_**-B**_**21**_ **vs b**_**1**_**-b**_**21**_**]**, 21 pairs of isomeric sphingolipids; [**IS]**, internal standard

**Fig. 1:**
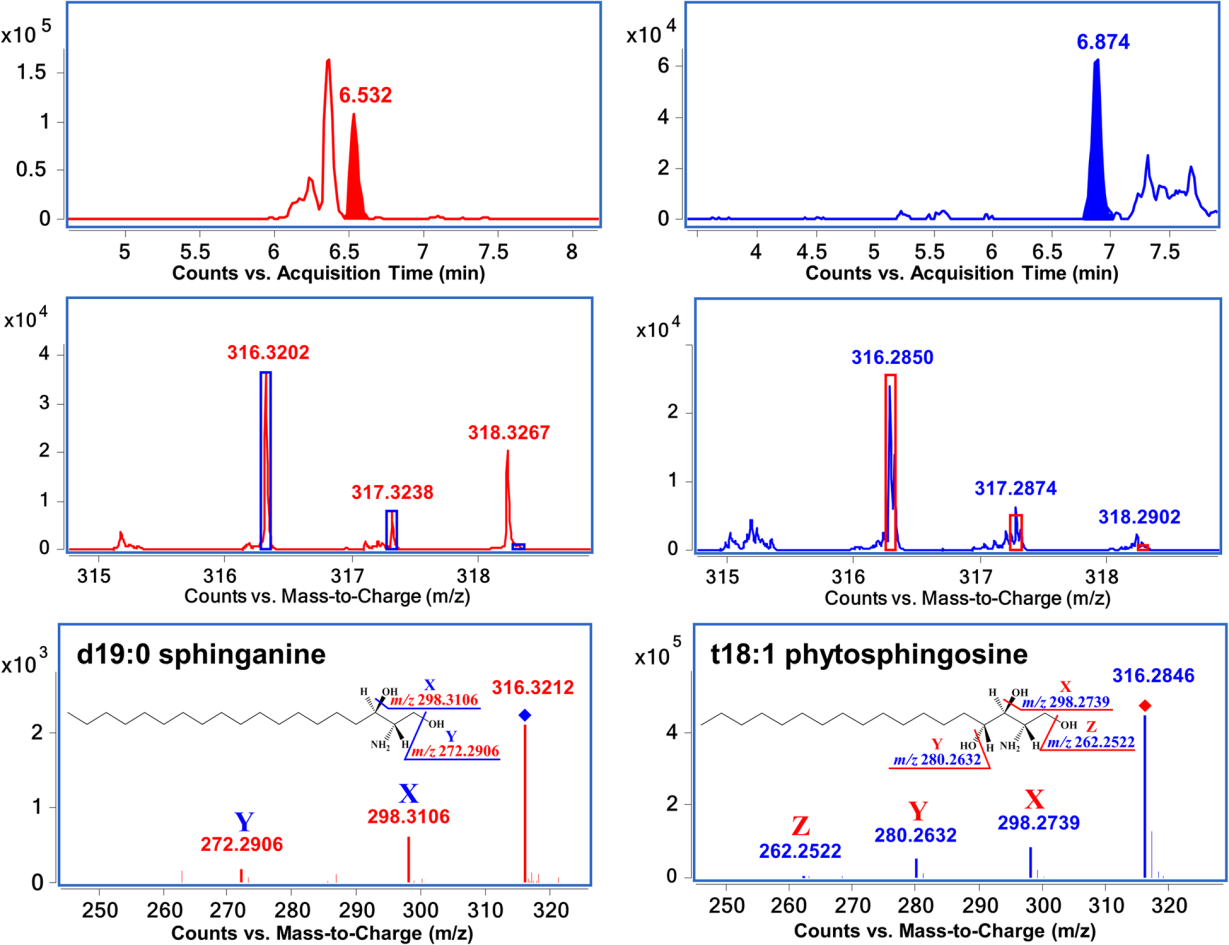
Differentiation of isobaric SPLs by high resolution mass spectrometry. Accurate mass and isotope distribution can distinguish two *m*/*z* 316 compounds, corresponding to d19:0 sphinganine (C_19_H_41_NO_2_) and t18:1 phytosphingosine (C_18_H_37_NO_3_), respectively. Typical ion fragments in MS/MS confirmed the identification

Interpretation of high resolution MS and MS/MS spectra of each identified ion, as well as searching against the latest database, allowed for the accurate identification of SPLs. For instance, isobaric lipids could be differentiated by the high-resolution mass spectrometry-based approaches. Two peaks yield *m*/*z* 316 ions, with accurate mass acquired by Q-TOF, *m*/*z* 316.3202 at 6.532 min and *m*/*z* 316.2850 at 6.874 min correspond to [C_19_H_41_NO_2_ + H]^+^ and [C_18_H_37_NO_3_ + H]^+^ respectively, facilitating assignment of sphinganine (Sa) d19:0 and phytosphingosine (PTSo) t18:1. Further fragmentation in MS/MS confirmed the identification, a consecutive loss of 3 hydroxy groups can be observed in the latter case, which is the characteristic cleavage of PTSo (Fig. 1).

**Fig. 2:**
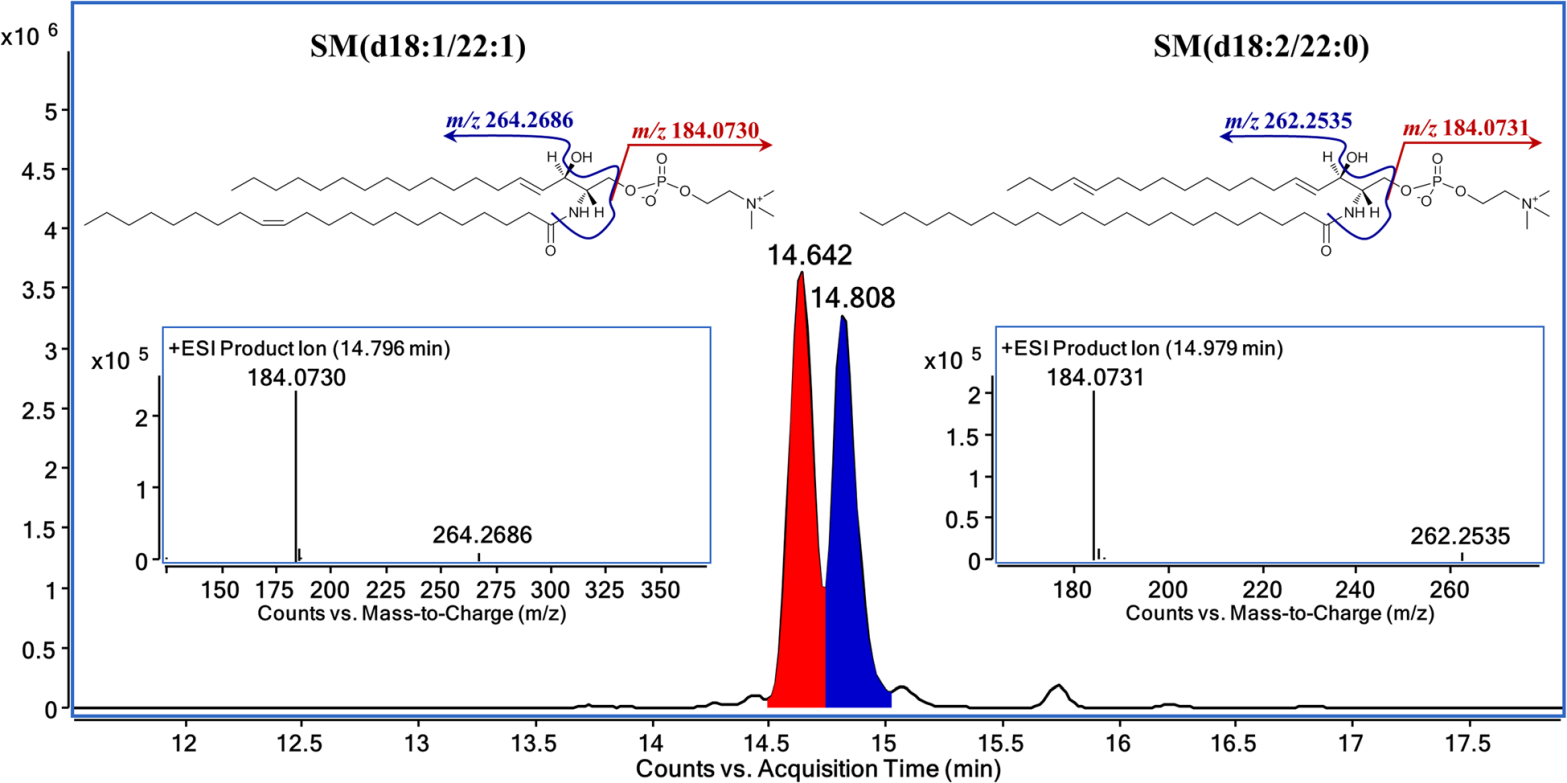
Differentiation of isomeric SPLs by accurate MS/MS. The extracted ion chromatogram of *m*/*z* 785.6423 at 5 ppm mass accuracy showed two peaks at 14.642 and 14.808 min. Targeted MS/MS of *m*/*z* 785.64 at respective time points gave distinct product ions corresponding to backbone of SM (d18:1/22:1) (*m*/*z* 264.3) and SM (d18:2/22:0) (*m*/*z* 262.3), providing evidence for the identification of these two species

A more realistic interference in the identification of SPLs is the isomeric species that have same number of atoms of each element, thus MS/MS fragment data with the assistance of optimized separation are essential for distinguishing the isomers. Take SM (d18:1/22:1) and SM (d18:2/22:0) as example, there are 2 peaks corresponding to *m*/*z* 785.65 in extracted ion chromatogram of TOF MS. In accurate MS/MS data acquired by Q-TOF, two characteristic fragments (264.3 & 262.3) respectively corresponding to the sphingoid base chain of SM (d18:1/22:1) and SM (d18:2/22:0) were observed (Fig. 2). The targeted ion pairs as well as complete chromatographic separation make the accurate MRM quantification of isomers possible.

Ceramides are prone to fragment into product ions corresponding to the sphingoid base backbone (e.g. *m*/*z* 262.25, 264.27, 266.28). In A549 QC samples, 29 Cers, including 20 dehydroceramides, 8 dihydroceramides (DHCers) and 1 phytoceramide (PTCer), were identified by comparing the MS information and retention time with those of SPLs in our previous study [10, 11]. Most Cers detected in the samples were with a d18:1 sphingoid backbone and the carbon number of N-acyl side chain varied from 14 to 26. A new dihydroceramide DHCer (d18:0/24:2), and Cer (d18:2/26:2), a dehydroceramide with high degree of unsaturation and long N-acyl chain, have been characterized for the first time to the best of our knowledge.

SM is the most multitudinous subclass of SPLs in A549 and A549T. Based on the exact mass in TOF MS and characteristic product ions obtained by Q-TOF MS/MS, a total of 56 SMs, including 38 dehydrosphingomyelins and 18 dihydrosphingomyelins (DHSMs), were unambiguously identified. All these SMs were characterized with a C18 sphingoid base chain, among which d18:1 type takes the largest proportion. In the N-acyl side chain, the number of carbon ranged between 14 and 26, with an unsaturation degree up to 5. Notably, all the DHSMs with 21 or less carbons in the N-acyl chain are fully saturated, while the others (with more than 21 carbons in the N-acyl chain) can be detected together with their corresponding de-hydrogen form. Three highly unsaturated SMs (total unsaturation degree no less than 4) including SM (d18:1/24:3), SM (d18:2/24:2) and SM (d18:2/24:3), have been detected in the QC sample of A549 & A549T cells.

Hexose-linked glycoceramide including galactosylceramide (GalCer) and glucosylceramide (GluCer) were represented as HexCer. All the 6 HexCers and 6 LacCers were found with d18:1 sphingoid base backbone. Only one HexCer with N-acyl chain in odd carbon number, HexCer (d18:1/23:0) was identified in A549. Notably, among all the HexCers and LacCers, only d18:1/24:1 species were identified as glycoceramides with unsaturated N-acyl fatty chain.

Seventeen sphingoid bases as well as the analogs were also successfully identified. The carbon number ranging from 14 to 19 and the degree of unsaturation falls between 0 and 2. Two PTSo with 3 hydroxyl groups, PTSo t19:2 and PTSo t16:1, have been discovered for the first time.

### Quantitation of sphingolipids in A549 and A549T cells

**Fig. 3:**
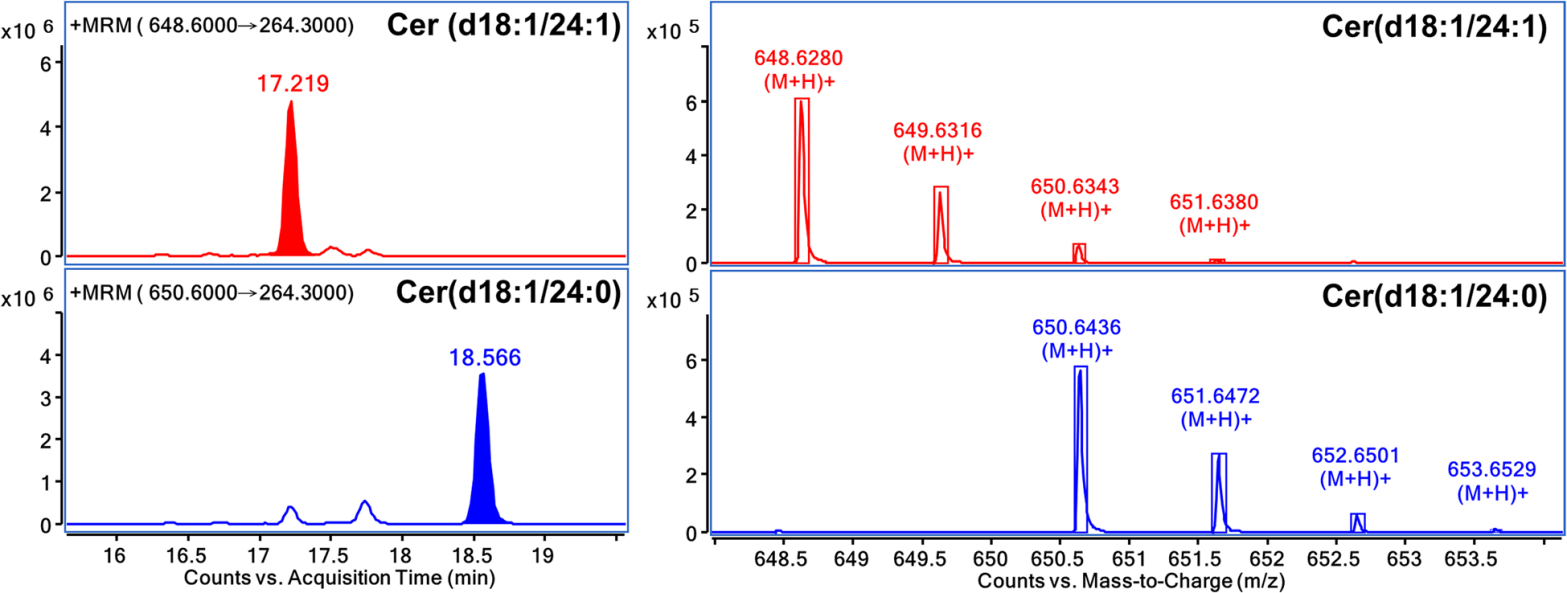
Differentiation of isotopic SPLs by accurate MS together with complete separation. Cer (d18:1/24:1) (t_R_ = 17.219 min) yields precursor ions at *m*/*z* 648.6280 and *m*/*z* 650.6343, the latter one is the [M + 2] isotopic ion which will interfere with the precursor ion of Cer (d18:1/24:0) (t_R_ = 18.566 min) at *m*/*z* 650.6436. The mass differentiation cannot be distinguished by QQQ. If the two peaks cannot be completely separated by LC, the quantification result of Cer (d18:1/24:0) will be artificially high

MRM mode of UHPLC-QQQ MS could provide accurate and sensitive approach under a wide range for quantitative analysis of SPLs. As the accuracy of triple-quadruple is about 0.1 Da, the quantification of SPLs cannot be accurately achieved merely with a QQQ analyzer, especially when suffering the isotopic interferences. Every unsaturated SPL could be recognized as an isotope of another one with the same characteristic backbone but less degree of unsaturation. For instance, if the LC separation is incomplete, the content of Cer (d18:1/24:0) will be artificially high due to the interference of Cer (d18:1/24:1) (Fig. 3). In this study, based on UHPLC complete separation and Q-TOF comprehensive profiling, accurate quantification was accomplished by eliminating the isotopic interference. By using the UHPLC-QQQ MS method with the optimized MRM parameters, a total of 75 species out of 114 identified SPLs were quantified in A549 and A549T cells, respectively. The amounts of these SPLs were quantified by comparing with the foregoing mentioned ISs.

**Fig. 4:**
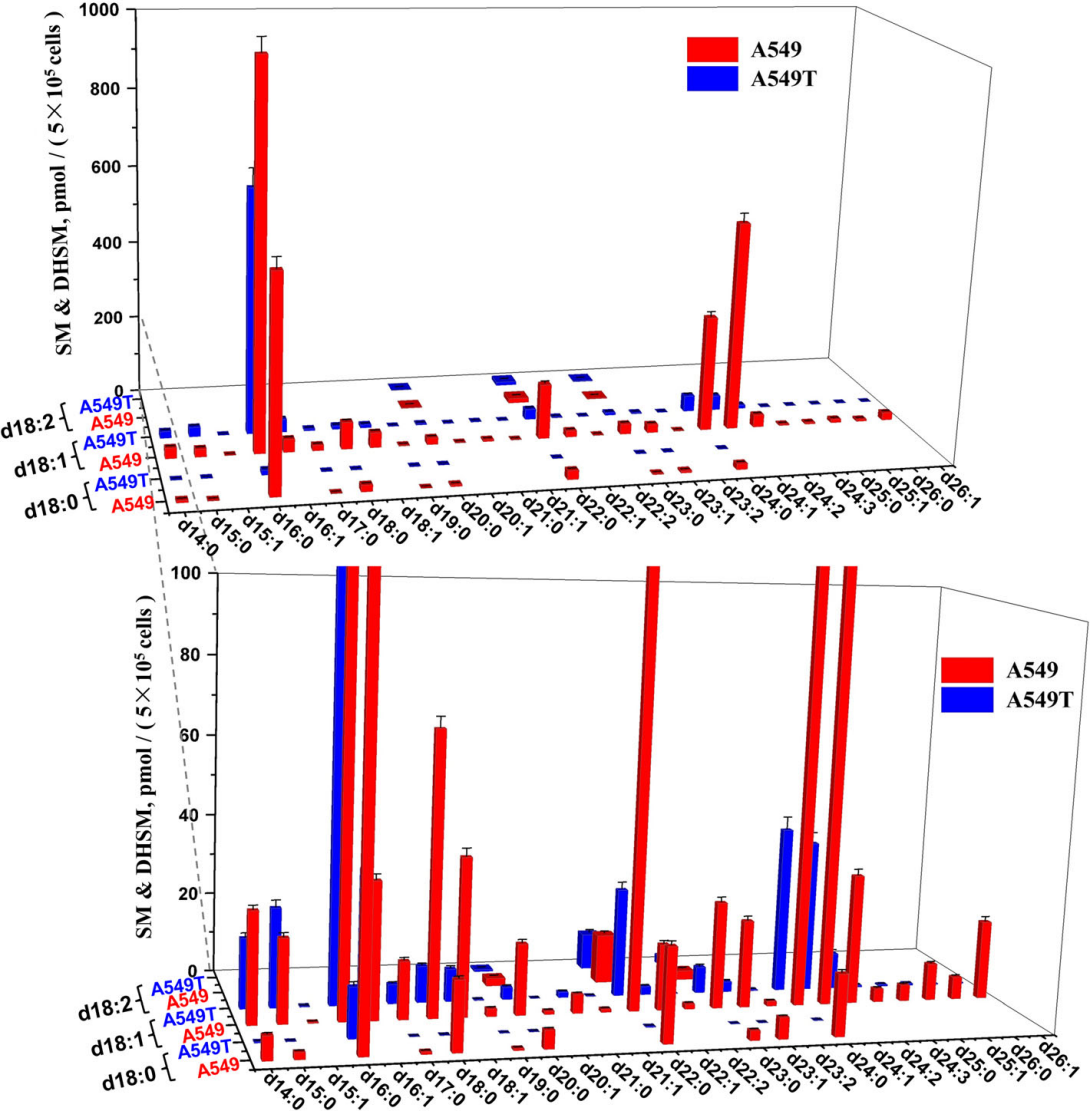
Content of SM and DHSM in A549 and A549T. The X and Z axis represent the compose of fatty acid acyl chain and backbone chain, respectively. Comparisons were performed by the non-parametric Mann-Whitney test. Most SMs and DHSMs showed statistical significance between A549 and A549T (*P* < 0.0001), except for SM (d18:1/16:1) and SM (d18:2/23:0) (*P* > 0.05)

**Fig. 5:**
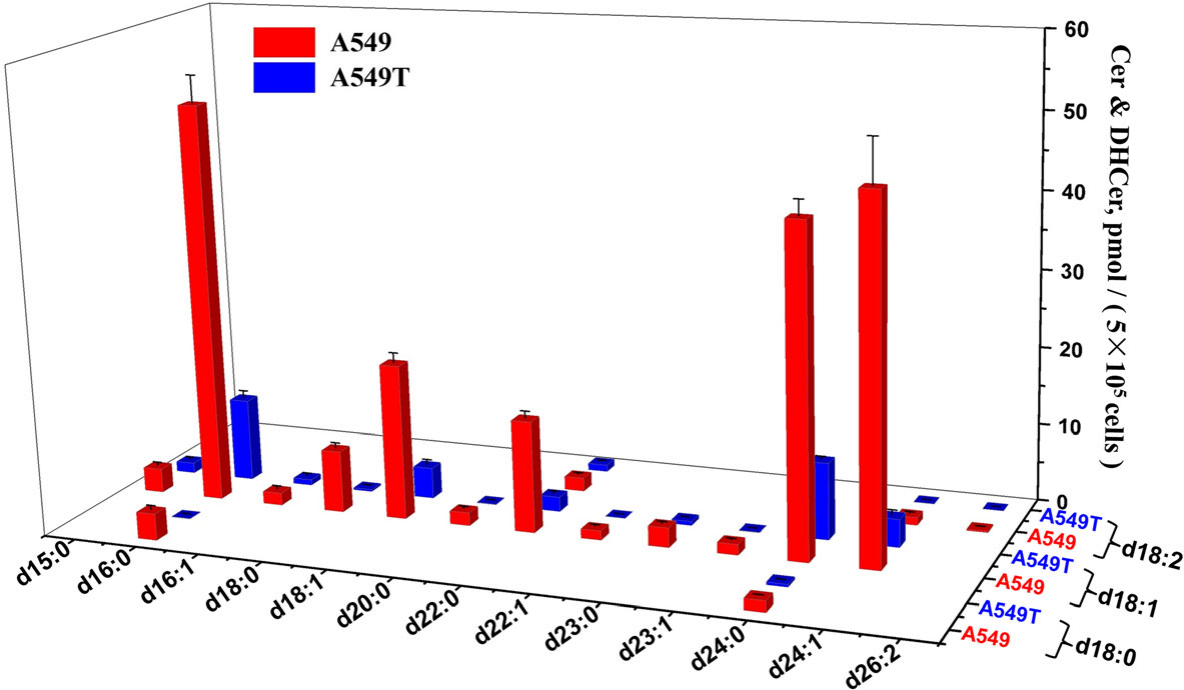
Content of Cer and DHCer in A549 and A549T. The X and Z axis represent the compose of fatty acid acyl chain and backbone chain, respectively. Comparisons were performed by the non-parametric Mann-Whitney test. All Cers and DHCers showed statistical significance between A549 and A549T (P < 0.0001)

**Fig. 6:**
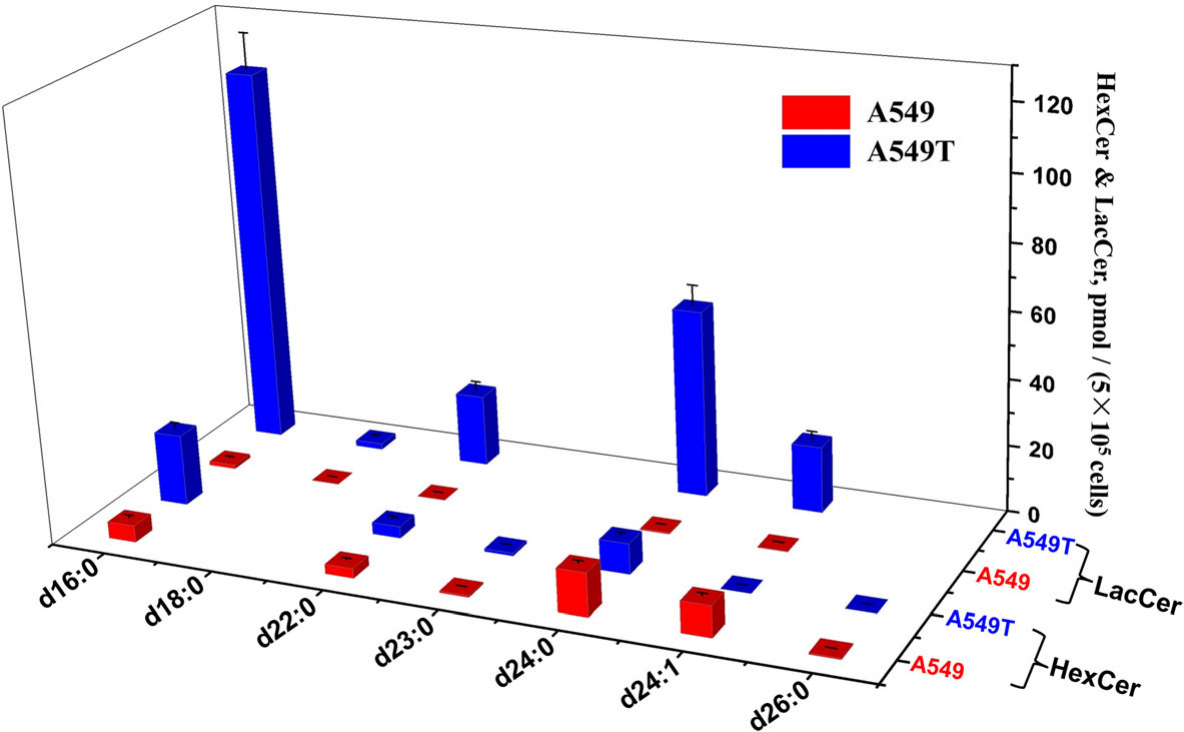
Content of HexCer and LacCer in A549 and A549T. The X axis represents the compose of fatty acid acyl chain of the d18:1 HexCer and d18:1 LacCer. Comparisons were performed by the non-parametric Mann-Whitney test. All HexCers and LacCers showed statistical significance between A549 and A549T (P < 0.0001), except for HexCer (d18:1/22:0) (*P* < 0.01) and HexCer (d18:1/23:0) (*P* < 0.001)

**Fig. 7:**
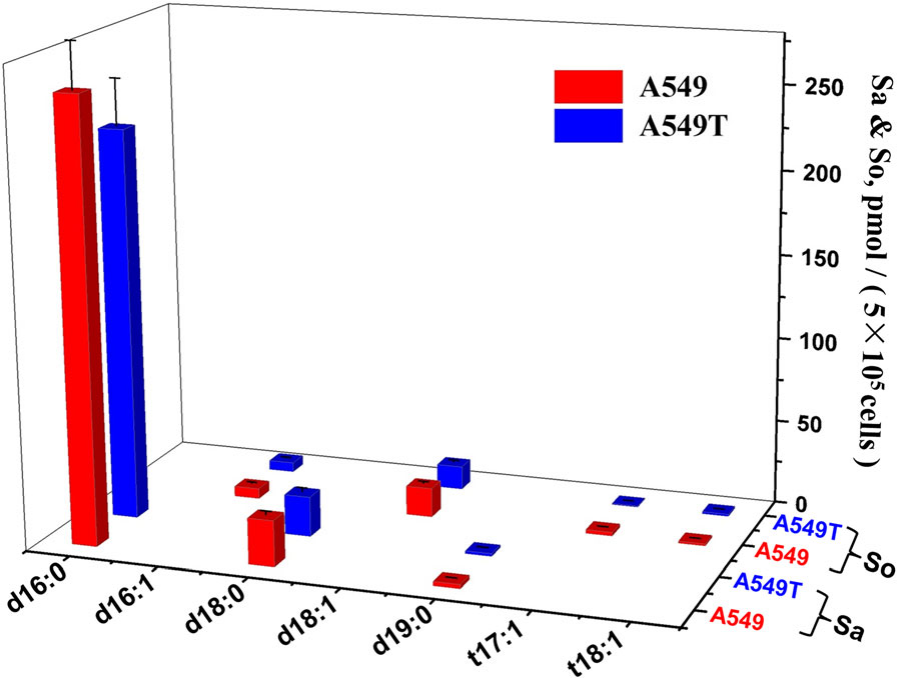
Content of sphingoid base in A549 and A549T. Comparisons were performed by the non-parametric Mann-Whitney test. All sphingoid bases showed statistical significance between A549 and A549T (*P* < 0.0001), except for Sa (d16:0) (*P* < 0.01), So (d16:1) (*P* > 0.05) and So (t18:1) (*P* > 0.05)

The quantitative results indicated that SMs account for the majority of all the SPLs in A549 and A549T, among which SMs with C16/C18/C22/C24 N-acyl side chain took the largest proportion of the total content. SMs with d18:1 sphingoid backbone are the most dominant species, which take 27 out of all the 41 quantified SMs (Fig. 4). For some SMs with high unsaturation degree or long N-acyl chain, the content is extremely low which cannot reach the limit of quantitation (LOQ). Figure 5 shows quantification data of 17 Cers. In general, the amounts of various Cers are significantly higher in A549 rather than those in A549T. Similar to SM, d18:1 Cers with C16/C18/C22/C24 N-acyl side chain showed relative high levels in both A549 and A549T, which take most proportion of Cer. LacCers and HexCers were only found with d18:1 sphingoid base backbone. All the 6 LacCers showed higher intensity in A549T than that in A549. But HexCer showed a species-dependent trend, HexCer d18:1/16:0, HexCer d18:1/22:0 and HexCer d18:1/23:0 increased in A549T, while HexCer d18:1/24:0, HexCer d18:1/24:1 and HexCer d18:1/26:0 decreased (Fig. 6). The overall content of sphingoid bases was similar in both cell types, Sa d16:0 was found with the highest intensity (Fig. 7). The relative abundance of each SPL varied greatly, but SPLs with N-acyl chain length of C16 and C24, respectively, are the most abundant species within each subclass.

**Fig. 8:**
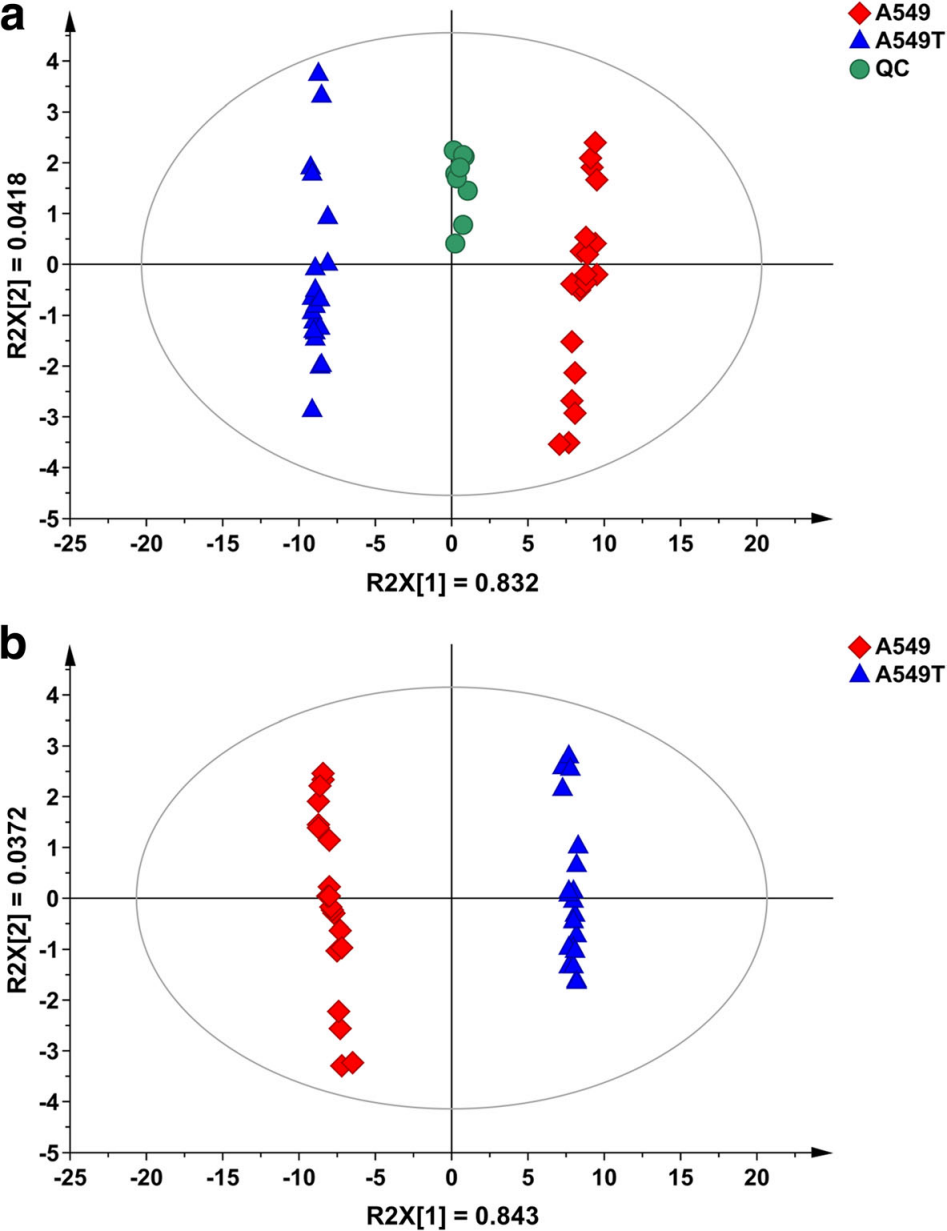
Principal component analysis and partial least squares discriminant analysis projecting scatter plots. **a** PCA [R^2^X (cum) = 0.874, Q^2^ (cum) = 0.845] score plots based on the content of SPLs obtained from A549 (red, diamond), A549T (blue, triangle), and QC (green, circle) groups; **b** PLS-DA [R^2^X (cum) = 0.880, R^2^Y (cum) = 0.999, Q^2^ (cum) = 0.998] score plots based on the content of SPLs obtained from A549 (red, diamond) and A549T (blue, triangle) groups

PCA was used for the overview of SPL dataset and the spotting of outliers, and thereby pick out trends of grouping or separation. It was performed to visualize general clustering among A549, A549T and QC groups [R^2^X (cum) = 0.874, Q^2^ (cum) = 0.845; Fig. 8a]. Supervised PLS-DA was used to further study the differences between A549 and A549T and to select potential biomarkers. In PLS-DA, the result of model showed the performance statistics of R^2^X (cum) = 0.880, R^2^Y (cum) = 0.999 with an excellent prediction parameter Q^2^ (cum) = 0.998, and the score plot showed good visual separation between A549 and A549T groups as well (Fig. 8b). A total of 57 potential biomarkers were identified according to scattering-plot and the VIP value (Table 2), among which most of them are SM and Cers. SM (d18:0/18:0) showed the largest decline in A549T, the content in decreased from 17.0 to 0.10 pmol/(5 × 10^5^cells), that markedly contributes to the classification.

**Table 2:** Quantification of SPLs (VIP > 1) in A549 and A549T

SPLs	Content (pmol/5*10^5^ cells)		ChangeA549T vs A549	*p* value	VIP
	A549 (*n* = 20)	A549T (*n* = 20)			
SM (d18:2/20:0)	1.96 ± 0.16	0.53 ± 0.07	↓	< 0.001	1.07408
SM (d18:1/26:1)	19.8 ± 1.22	0.34 ± 0.04	↓	< 0.001	1.08696
SM (d18:1/26:0)	5.67 ± 0.31	0.20 ± 0.02	↓	< 0.001	1.08775
SM (d18:1/25:1)	9.28 ± 0.43	0.45 ± 0.05	↓	< 0.001	1.08860
SM (d18:1/25:0)	4.09 ± 0.23	0.42 ± 0.04	↓	< 0.001	1.08699
SM (d18:1/24:3)	3.34 ± 0.26	0.47 ± 0.07	↓	< 0.001	1.08120
SM (d18:1/24:2)	32.5 ± 1.55	8.99 ± 0.87	↓	< 0.001	1.08501
SM (d18:1/24:1)	542 ± 23.8	37.9 ± 2.59	↓	< 0.001	1.08879
SM (d18:1/24:0)	298 ± 13.0	41.5 ± 3.16	↓	< 0.001	1.08829
SM (d18:1/23:2)	1.12 ± 0.11	0.25 ± 0.04	↓	< 0.001	1.06488
SM (d18:1/23:1)	21.6 ± 1.08	2.58 ± 0.30	↓	< 0.001	1.08745
SM (d18:1/23:0)	26.4 ± 1.26	6.57 ± 0.51	↓	< 0.001	1.08618
SM (d18:1/22:2)	1.25 ± 0.12	0.15 ± 0.02	↓	< 0.001	1.07067
SM (d18:1/22:1)	16.4 ± 0.81	2.07 ± 0.18	↓	< 0.001	1.08760
SM (d18:1/22:0)	142 ± 6.17	26.9 ± 1.78	↓	< 0.001	1.08783
SM (d18:1/21:1)	0.82 ± 0.07	0.15 ± 0.02	↓	< 0.001	1.07308
SM (d18:1/21:0)	4.66 ± 0.25	1.39 ± 0.12	↓	< 0.001	1.08216
SM (d18:1/20:1)	0.87 ± 0.13	0.06 ± 0.00	↓	< 0.001	1.06102
SM (d18:1/20:0)	17.6 ± 0.86	3.08 ± 0.31	↓	< 0.001	1.08689
SM (d18:1/19:0)	1.94 ± 0.20	0.21 ± 0.03	↓	< 0.001	1.07588
SM (d18:1/18:1)	39.0 ± 1.99	8.13 ± 0.58	↓	< 0.001	1.08637
SM (d18:1/18:0)	69.5 ± 2.72	9.07 ± 0.54	↓	< 0.001	1.08896
SM (d18:1/17:0)	14.1 ± 0.72	4.76 ± 0.39	↓	< 0.001	1.08307
SM (d18:1/16:0)	982 ± 38.5	629 ± 23.5	↓	< 0.001	1.06108
SM (d18:1/14:0)	27.3 ± 1.04	17.2 ± 0.65	↓	< 0.001	1.06732
SM (d18:0/24:0)	15.3 ± 0.78	0.11 ± 0.02	↓	< 0.001	1.08843
SM (d18:0/23:0)	2.45 ± 0.15	0.02 ± 0.00	↓	< 0.001	1.08694
SM (d18:0/22:0)	23.3 ± 1.05	0.23 ± 0.04	↓	< 0.001	1.08900
SM (d18:0/20:0)	4.59 ± 0.24	0.08 ± 0.01	↓	< 0.001	1.08808
SM (d18:0/19:0)	0.49 ± 0.07	0.01 ± 0.00	↓	< 0.001	1.05519
SM (d18:0/18:0)	17.0 ± 0.55	0.10 ± 0.03	↓	< 0.001	1.09013
SM (d18:0/17:0)	0.65 ± 0.07	0.06 ± 0.01	↓	< 0.001	1.07030
SM (d18:0/16:0)	545 ± 28.7	12.3 ± 0.94	↓	< 0.001	1.08809
SM (d18:0/15:0)	1.74 ± 0.16	0.10 ± 0.02	↓	< 0.001	1.08041
SM (d18:0/14:0)	5.83 ± 0.40	0.28 ± 0.03	↓	< 0.001	1.08565
Cer (d18:2/24:1)	1.01 ± 0.21	0.03 ± 0.01	↓	< 0.001	1.04397
Cer (d18:1/24:1)	46.1 ± 5.83	3.60 ± 0.44	↓	< 0.001	1.07110
Cer (d18:1/24:0)	42.0 ± 2.08	9.83 ± 0.41	↓	< 0.001	1.08653
Cer (d18:1/23:1)	1.37 ± 0.22	0.06 ± 0.01	↓	< 0.001	1.06057
Cer (d18:1/23:0)	2.54 ± 0.27	0.53 ± 0.09	↓	< 0.001	1.06941
Cer (d18:1/22:1)	1.20 ± 0.27	0.04 ± 0.01	↓	< 0.001	1.03542
Cer (d18:1/22:0)	14.4 ± 0.94	1.90 ± 0.39	↓	< 0.001	1.08443
Cer (d18:1/20:0)	1.69 ± 0.33	0.07 ± 0.02	↓	< 0.001	1.04721
Cer (d18:1/18:1)	20.1 ± 1.33	4.03 ± 0.49	↓	< 0.001	1.08187
Cer (d18:1/18:0)	8.03 ± 0.72	0.26 ± 0.04	↓	< 0.001	1.08142
Cer (d18:1/16:0)	52.0 ± 3.59	10.7 ± 0.98	↓	< 0.001	1.08243
Cer (d18:1/15:0)	3.14 ± 0.46	1.34 ± 0.30	↓	< 0.001	1.00100
Cer (d18:0/24:0)	1.44 ± 0.11	0.34 ± 0.04	↓	< 0.001	1.07587
Cer (d18:0/16:0)	3.46 ± 0.25	0.07 ± 0.01	↓	< 0.001	1.05883
HexCer (d18:1/26:0)	0.41 ± 0.10	0.04 ± 0.02	↓	< 0.001	1.00780
HexCer (d18:1/24:1)	9.25 ± 0.97	0.02 ± 0.00	↓	< 0.001	1.07959
HexCer (d18:1/16:0)	5.06 ± 0.89	21.6 ± 2.10	↑	< 0.001	1.07118
LacCer (d18:1/24:1)	0.22 ± 0.05	19.9 ± 2.17	↑	< 0.001	1.07014
LacCer (d18:1/24:0)	0.37 ± 0.03	56.4 ± 3.16	↑	< 0.001	1.07766
LacCer (d18:1/22:0)	0.06 ± 0.01	21.6 ± 2.85	↑	< 0.001	1.07258
LacCer (d18:1/16:0)	1.15 ± 0.55	115 ± 11.6	↑	< 0.001	1.08013
So (t17:1)	2.21 ± 0.17	0.62 ± 0.08	↓	< 0.001	1.07483

## Discussion

Using the sphingolipidomic approach, we obtained the detailed sphingolipid profiles for human lung adenocarcinoma cell A549 and its taxol resistant strain A549T, and then performed quantification. We found A549 and A549T share all the same species of SPLs, among which SM (dehydrosphingomyelin and DHSM), Cer (dehydroceramide, DHCer and PTCer), HexCer, LacCer, and sphingoid base were identified as the major SPLs. In contrast to normal A549, decreasing levels of Cer and SM concomitant with increasing of glycosphingolipids represent the main SPL metabolic profile of A549T. Totally 35 SMs, 14 Cers, 3 HexCers, 4 LacCers, and 1 sphingosine are recognized as metabolic pathway related biomarkers.

**Fig. 9:**
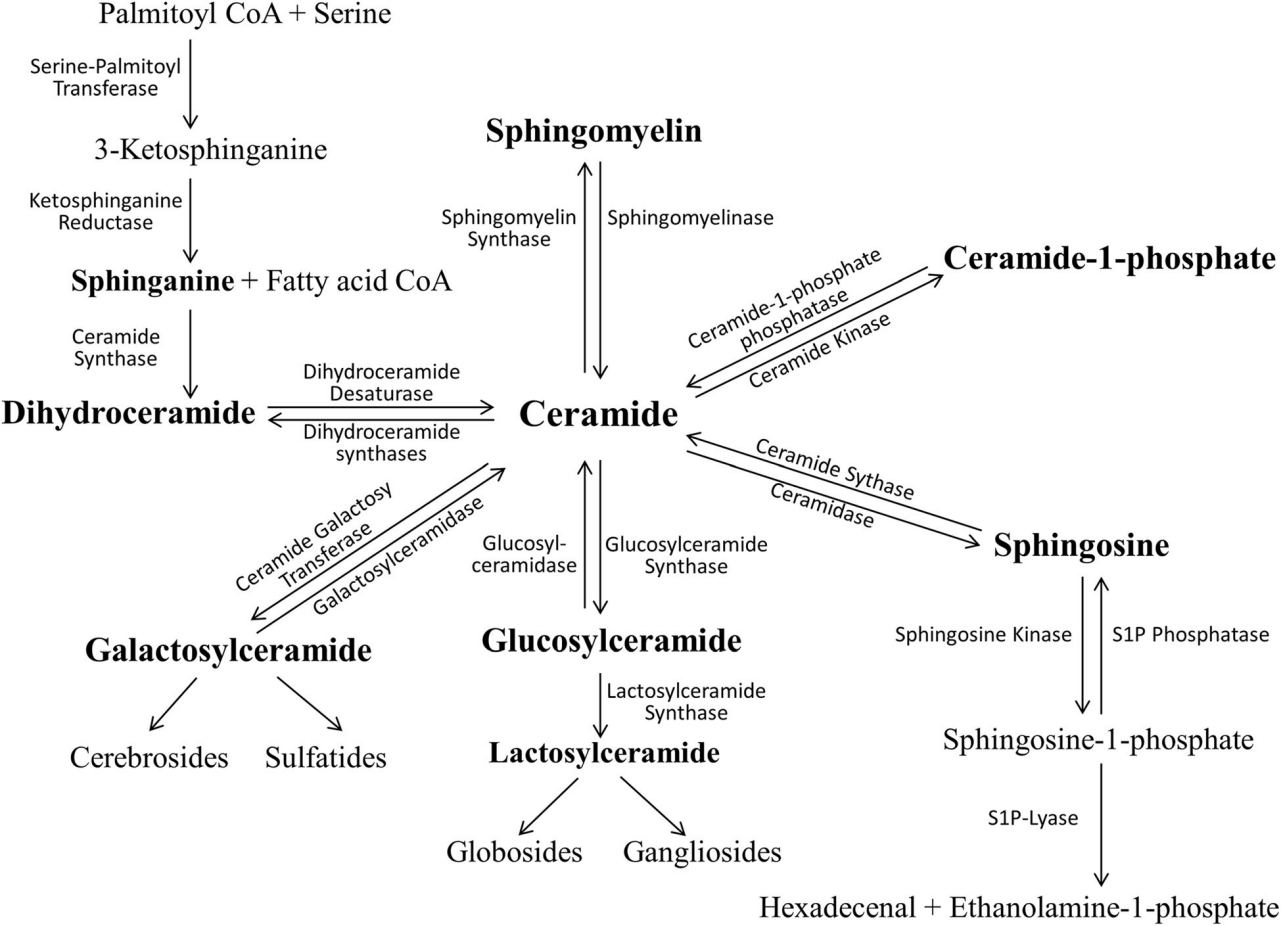
Biosynthesis and metabolism pathway of sphingolipids

Cer is the basic SPL structural unit which balances cell growth and death by inducing apoptosis [12], and its definite efficacy in promoting apoptosis in A549 cells has been well studied [7]. It is noteworthy that Cers can be classified into SM-hydrolyzed and de novo-synthesized. The former is well known as triggering apoptotic death signaling in many cell types, while the specific role of the latter one seems important to tumor survival [13]. In human ovarian carcinoma cell line CABA I, anti-cancer drugs including taxol have been reported to activate SMase to generate Cer, which acts as a second messenger in triggering apoptosis [14]. While in lung carcinoma cells, the tumor tissues produce large amounts of both dihydroceramide and ceramide through the de novo synthesis pathway, but not through SM hydrolysis [13]. More relevantly, treatment of A549 cells with gemcitabine was demonstrated to increase Cer levels via the activation of de novo synthesis [15]. In the case of study of A549T in this paper, both Cer and SM levels were much lower than their levels in taxol-sensitive A549 cells, which indicates that the decrease of Cer may not attribute to “activating the SM pathway” as our previous study in A2780T [11]. Furthermore, DHCer was decreasing accompanied with Cer, which revealed the mechanism of taxol-resistance in A549T could be explained as “inhibiting the *de novo* synthesis pathway”. (Fig. 9).

Both Cer and its catabolite sphingosine as negative regulators of cell proliferation could promote apoptosis, and the role of sphingosine as a messenger of apoptosis is of importance [16]. In small cell lung cancer (SCLC), multidrug-resistance-associated protein (MRP) contributes to the drug resistance, and pro-apoptotic SPLs (Cer and sphingosine) could further induce apoptosis overcome or bypass MRP-mediated drug resistance [17]. In non-small cell lung cancer (NSCLC) including A549, sphingosine kinase 2 (SphK2) is proposed to be the key regulator of sphingolipid signaling which may contribute to the apoptosis resistance [18]. Inhibition of SphK2 can enhance the apoptosis of NSCLC cells, and it will certainly result in an increase of the substrate sphingosine. In A549T, all sphingosines showed consistent trend of decrease comparing to A549. It’s known that sphingosine in mammalian cells is not synthesized de novo but it is generated from ceramides by ceramidases [19]. Thus, we can deduce that in A549T the concomitant decrease of sphingosine and Cer may be the result of activation of SphK2, which leads to the inhibition of apoptosis in taxol resistant strain.

Besides Cer and SM, glycosphingolipids including HexCers (GalCers & GluCers) and LacCers account for a large proportion of biomarkers in A549T. It has been observed that glucosylceramide synthase is up-regulated after drug intervention and suggests that glycolipids may be involved in chemotherapy resistance [2]. For decades, GluCer has been found to increase in the resistant cancer cells [20], suggesting that glycosylation plays an important role in evading Cer induced apoptosis. Glycosphingolipids have recently been reported as transactivating multidrug resistance 1/P-glycoprotein (MDR1) and multidrug resistance-associated protein 1 (MRP1) expression which further prevents accumulation of ceramide and stimulates drug efflux [21]. Specifically, GalCer and LacCer were characteristically increased in taxol-resistant human ovarian carcinoma-derived KF28TX cells [22]. Moreover, GalCer was demonstrated to be the apoptosis protector, and its upregulation was also thought to attenuate the Cer-mediated apoptotic signals [23]. Our findings revealed a significant overall increase of glycosphingolipids in A549T, among which all LacCers showed a consistent tendency of increase, while HexCers (including GalCer and GluCer) showed a species-dependent trend. It should be noted that GluCer could be converted into LacCer under the influence of lactosylceramide synthase. Therefore the decreased species of HexCer might be GluCer. For instance, the decrease of HexCer (d18:1/24:1) resulted in the concomitant increase of LacCer (d18:1/24:1).

## Conclusions

Evidences suggest that tumor microenvironment including the sphingolipidome plays an important role in cancer drug resistance. So far to our knowledge, there is no sphingolipidomic study on taxol-resistant A549 human adenocarcinoma cell line. Based on the comprehensive identification and accurate quantification of SPLs, decreasing of Cer, SM and sphingosine concomitant with increasing of HexCer and LacCer have been characterized as the metabolic profile of A549T. It indicated that “inhibition of the *de novo* synthesis pathway” and “activation of glycosphingolipid pathway” played the dominant role for taxol-resistance, and the key enzymes related to the pathways may have been altered. These results provide evidence to unravel the mechanism of taxol resistance in A549T. The distinctive phenotype could facilitate clinical diagnosis of taxol-resistant adenocarcinoma and provide insights into targets for the development of new drug against taxol resistance.

## Abbreviations

A549T: Taxol-resistant strain of A549 cell
C1P: Ceramide-1-phosphate
Cer: Ceramide
DHCer: Dihydroceramide
DHSM: Dihydrosphingomyelin
HexCer: Hexosylceramide
LacCer: Lactosylceramide
QC: Quality control
SM: Sphingomyelin
SPL: Sphingolipid
